# MI (2-methyl-4-isothiazolin-3-one) contained in detergents is not detectable in machine washed textiles

**DOI:** 10.1186/s13601-017-0187-2

**Published:** 2018-01-05

**Authors:** Maja A. Hofmann, Ana Giménez-Arnau, Werner Aberer, Carsten Bindslev-Jensen, Torsten Zuberbier

**Affiliations:** 1 Department of Dermatology and Allergy, Charité-Universitätsmedizin Berlin, Charitéplatz 1, 10117 Berlin, Germany; 2 Department of Dermatology and Allergy Center, Odense Research Center for Anaphylaxis (ORCA), Odense University Hospital, University of Southern Denmark, 5000 Odense C, Denmark; 3 Department of Dermatology, Hospital del Mar, Universitat Autònoma, Barcelona, Spain; 4 Department of Dermatology and Venerology, Medical University of Graz, Auenbruggerplatz 8, 8036 Graz, Austria

**Keywords:** MI, Laundry detergent, Preservative, Contact allergy

## Abstract

**Background:**

European legislation has banned the preservative methylisothiazolinone (MI) from inclusion in leave-on cosmetics. However, the risk for allergic reactions depends on exposure. The aim of this study was to determine the risk of MI in laundry detergents for household machine washing.

**Methods:**

Different formulations of laundry detergents with commercial MI levels, up to one thousand ppm were used and three different types of clothes were washed in a normal household machine setting one time and 10 times. The level of MI was measured by HPLC.

**Results:**

While MI could be retrieved in the positive control of clothes drenched with washing powder but not washed afterwards, MI could not be detected in any specimen of clothes washed under household conditions. The detection limit was 0.5 ppm.

**Conclusion:**

It is important to discuss the difference of risk and hazard. While MI clearly is a high hazard as a strong contact allergen, the risk depends on exposure. Regarding the risk of exposure levels for the consumer to MI in clothes it can be stated that the use of MI in laundry detergents is safe for the consumer if these products are used according to the instructions in the normal household setting machine wash.

## Introduction

Methylisothiazolinone (MI) belongs to the group of isothiazolinones, together with methylchloroisothiazolinone (MCI) and benzisothiazolinone (BIT). MI is often used alone, but it is also common to use MCI and MI in combination as preservatives, firstly introduced in the 1980s in a fixed 3:1 (MCI/MI) combination for industrial use.

As MCI/MI have antimicrobial activities against gram+ and gram− bacteria, moulds and yeasts, they have been used as preservatives in cosmetic products and chemical products, such as in paints.

Contact allergy to methylisothiazolinone (MI) was commonly attributed to the usage in cosmetics but MI is also a common preservative in household detergents and water-based paint [1].

There is an ongoing important interest in MI as a preservative as the prevalence of sensitizations to MCI/MI and MI increased during the past years in Europe. In Germany, the sensitization rate to MCI/MI increased from 2.3% in 2009 to 3.9% in 2011 [2].

Up until the 1990s MI was only used as a preservative in cosmetics, in a concentration of 15 ppm in Europe, with a contact sensitization rate of about 2% with a stable trend. After the 2000, MI was introduced as a preservative also in non-cosmetics, chemical products (paint, inks, lacquers, varnishes, and cooling fluid). Firstly, occupational contact sensitizations to MCI/MI or MI were reported among painters and paint factory workers in particular [3]. MI was also allowed in cosmetic products up to a concentration of 100 ppm (Cosmetic Directive 2005/42/EC), as the antimicrobial effect is somewhat weaker than the MI/MCI combination, upon which an increase of non-occupational sensitization was noted [4].

As MI is volatile and can evaporate, it can also cause airborne contact dermatitis or asthmatic symptoms [5]. It was shown to be one of the most important contact allergens associated with airborne contact dermatitis such as components of epoxy resin systems and compositae [6].

Due to the increased sensitization rates to MI, the Scientific Committee on Consumer Safety of the European Commission (SCCS) has now banned this preservative from Leave-on cosmetics eventhough the range of the cosmetics are wide; including hair cosmetics, facial cosmetics, deodorants, sunscreens and wet wipes (baby wipes, moist tissues, moist toilet paper).

On the other hand, it should be noted that isothiazolinone derivates are used in the production of clothes and could be an independent source, although as of yet only occupational reactions to the preservative during the production have been described. No systematic review exists if remnants are found in the clothes. Furthermore, a combination of 5-chloro-2-methyl-4-isothiazolin-3-one and 2-methyl-4-isothiazolin-3-one was described to lead to occupational dermatitis in the nylon production and in spin finish [7, 8]. In both cases MI was used as a preservative not alone but in combination with methylchloroisothiazolinone. A further report has been published regarding this preservative combination called Acticide SPX, a 1.5% mixture of 5-chloro-2-methyl-1-4-isothiazolinone-3-one and 2-methyl-4-isothiazolin-3-one, flax spinners were sensitized by this combination occupationally [9].

The use of MI in rinse-off products (shampoos) has been limited to 15 ppm. As preservatives are necessary in many consumer products used in daily life, it is of high interest that alternatives should replace well-known preservatives without knowing the risk of their sensitization potential in the different product groups.

The aim of this study was to determine the risk of the consumer through using MI in laundry detergents based on the remnant of the preservative in the washed clothes. As of yet, no study exists if there is a risk that MI residues remain in washed clothes.

## Materials and methods

2-Methyl-4-isothiazolin-3-one (MI, purity ≥ 99%) was purchased from Fluka/Sigma (Buchs, Switzerland). Methanol LiChrosolv Gradient Grade for sample preparation and liquid chromatography was purchased from Merck (Darmstadt, Germany). Deionized water was produced via Merck-Millipore Milli-Q Avantage A10 facility. Ortho-phosphoric acid (p.A. 85%) and hydrochloric acid (p.A. 37%) were purchased from VWR International GmbH (Darmstadt, Germany). Disposable syringe filters (SRP-15 0.45 µm) were purchased from Sartorius (Goettingen, Germany).

Textile sample contained of wkf 10A (standard cotton), wfK 20A (polyester/cotton), wfK30 (polyester fibre), http://www.testgewebe.de/en/products/reference-and-standard-fabrics/.

As washing agent a commercially available universal detergent (Italian market) was used. Different concentrations of preservatives were added to this detergent: no preservative, 0.11% Acticide MBR1, 1.1% Acticide MBR1 (containing 9% of MI).

The washing process was performed with a Miele Novotronic W1734 household washing machine. A colour program with 20 °C with 3.5 kg laundry and 75 ml of washing agent was used. The textile samples were mixed with normal dirty clothes. Normal urban tap water was used and 4 SBS 2004 as ballast load were added (http://www.testgewebe.de/en/products/sbl/sbl-2004/).

The textile samples were dried on the line and washed either one time or 10 times. In the case of 10 times, the above-mentioned washing process was repeated the same way.

As positive control the same Textile samples of wkf 10A (cotton wool), wfK 20A (polyester fibre, cotton wool), wfK30 (polyester fibre) were drenched with the three washing powders with different concentrations of MI but not washed afterwards.

Approximately 2 g of each textile sample were cut into pieces of 1–2 cm^2^ and transferred into a glass vessel. Afterwards, 25 mL of a mixture containing 95% of diluted o-phosphoric acid (0.025%) and 5% of methanol (V/V) were added. The samples were extracted for 10 min using an ultrasonic bath. After extraction, an aliquot was filtered and used for HPLC analysis. The HPLC system was calibrated via MI calibration solutions ranging from 12 µg/L to 2.4 mg/L. For that purpose, the standard substance MI was first dissolved in methanol (30 mg in 100 mL) and afterwards diluted appropriately using a mixture containing 95% of diluted o-phosphoric acid (0.025%) and 5% of methanol (V/V).

The HPLC instrument was supplied by Waters (Milford, USA) and consisted of the modules Acquity UPLC™ BSM, Acquity UPLC™ SM, Acquity UPLC™ CM and Acquity UPLC™ PDA. For chromatographic separation, a Waters Acquity UPLC™ RP18 column (2.1 × 100 mm) was used, maintained at 40 °C in the column compartment. The injection volume was set to 10 µL. Gradient elution was performed using 0.25% *o*-phosporic acid (eluent A) and methanol (eluent B) at a flow rate of 0.4 mL/min. The analyte MI was detected at a wavelength of 274 nm and quantified using external standard calibration.

## Results

**Fig. 1:**
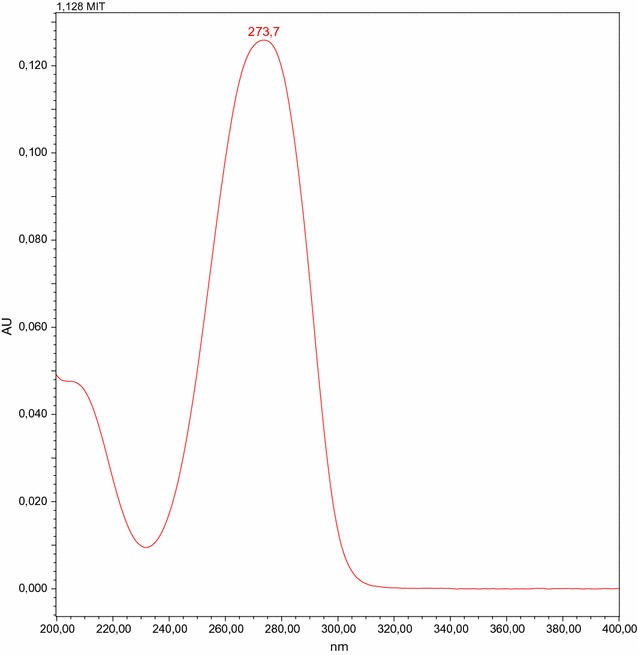
UV-spectrum of MI. Chromatographically analysis of MI, the maximum peak is seen at 273.3 nm

**Fig. 2:**
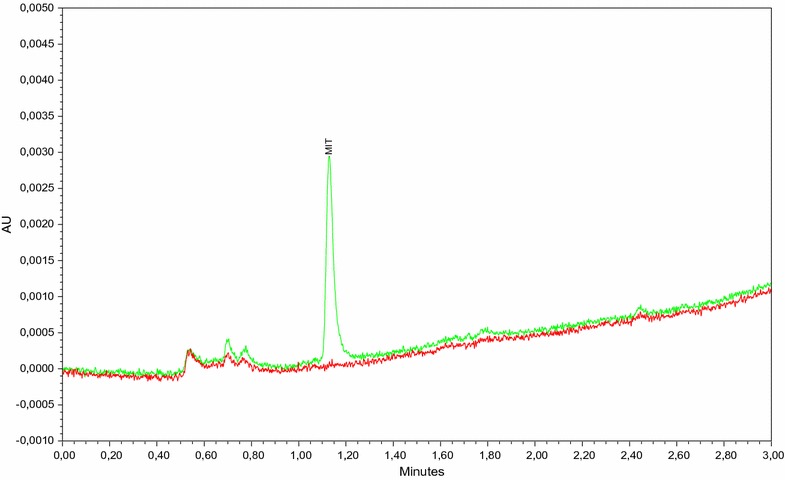
Chromatogram overlay of washed cotton swatch without MI and with MI. Proof of concept, validation of methodology. Washed cotton without MI (red) and with 0.9 µg/g MI (green), MI can be measured chromatographically in cotton wool samples

In the first step, it could be shown that 2-methyl-4-isothiazolin-3-one can be separated from interfering matrix compounds by chromatographic analysis. The analysis was run through with replicates of a cotton fabric (CO), washed one time and washed 10 times. The UV-spectrum of MI is shown in Fig. 1. Figure 2 shows the spiked washed cotton without and with 0.9 µg/g MI. It could be clearly shown that MI can be measured chromatographically in cotton wool samples.

For calibration, it could be shown that the calibration curve for 2-methyl-4-isothiazolin-3-one is linear in the calibration range of interest (R > 0.9999). System precision was determined by a sixfold injection of a calibration standard. The relative standard deviation (RSD) for 2-methyl-4-isothiazolin-3-one was determined to be 0.5%.

For determination of method precision, six replicates each of a cotton fabric (Co), polyester fabric and blended fabric (cotton with polyester) were spiked with 18 and 1.8 µg 2-methyl-4-isothiazolin-3-one. After sample analysis, a relative standard deviation of 1.0–1.4% was determined for the 18 µg spiking level, and 6–10% for the 1.8 µg spiking level. For determination of accuracy, the values obtained for method precision were used. A recovery rate of 97.9–100% was determined for the 18 µg spiking level, and 72.3–88.7% for the 1.8 µg spiking level.

The methodology has a detection limit of 0.098 µg of 2-methyl-4-isothiazolin-3-one related to 25 mL of extraction solution. Due to the sample preparation, this is equivalent to 0.05 µg/g related to the weighed fabric sample.

In experiments with machine washed cloth it could be shown that MI could not be detected either in cotton wool samples, or in polyester fibre or in polyester fibre/cotton wool samples after washing (Table 1). Furthermore there was no difference in the detection of MI regarding one washing or ten washes. This shows that there is no enrichment of potential residues after repeated washing. Even after using an artificially MI enriched detergent containing 1000 ppm MI which is far above the level found in commercialized products, there was no detection of MI in the chromatogram, after 1 wash or 10 washes.

**Table 1:** MI-residue analysis of laundry fabrics after washing

Products measured		PES washed		Co/PES washed		Co washed	
		1×	10×	1×	10×	1×	10×
w/o preservation (WLHUL1604500)	< 1 ppm	Not detectable	Not detectable	Not detectable	Not detectable	Not detectable	Not detectable
100 ppm MI (WLHUL1604600)	100 ppm	Not detectable	Not detectable	Not detectable	Not detectable	Not detectable	Not detectable
1000 ppm MI (WLHUL1604700)	1000 ppm	Not detectable	Not detectable	Not detectable	Not detectable	Not detectable	Not detectable

MI detection in PES, Co/PES and CO washed one time or ten times in different MI concentration (< 1, 100 and 1000 ppm). MI, 2-methyl-4-isothiazolin-3-one; Co, cotton fabric; PES, polyester fabric; co/PES, blended fabric (cotton with polyester), detection limit < 0.05 ppm

## Discussion

The increasing incidence of MI contact allergy is evident and is alarming.

In early years, sensitization levels were stable as MI was only used in cosmetics in combination with MCI and a low concentration up to 15 ppm. However, after the year 2000, the sensitization rate started to rise after published expert opinion suggesting that 100 ppm was safe. Furthermore, MI was used more repeatedly for occupational purposes due to workplace use of hygiene (healthcare) and beauty products (hairdressers and beauticians) together with water-based paints and other aqueous solutions such as cutting fluids [10]. Until now, MI has been used in a wide range of cosmetic products as well as Rinse-off and Leave-on cosmetics. Sensitization cases were attributed to Leave-on body products, like creams or lotions [11] and nowadays there is an increasing number of reports induced by rinse-off products containing MI [12–14]. Therefore, MI was banned as a preservative in leave-on/on products in 2013. It is also known that in patients with sensitization to MI, the usage of rinse-off products can elicit contact allergic symptoms in dosages of 100 ppm and less frequently also in lower dosages of 50 ppm [12] but the overall frequency is less high than seen with leave-on products.

Warburton et al. [15] tested with very high concentrations of 2000 ppm (0.2% as used in the British standard) and could only identify 3 patients who had developed eczema between July and December 2014, attributing it to rinse-off products.

This shows that the potent allergenic potential of MI has to be seen in the context of true exposure in different products and especially in products outside the cosmetics range such as laundry detergents. The data of this study revealed two important findings regarding this point. The first finding is that MI in laundry detergents is completely washed out after using a normal household machine washing. This is important regarding for both a NOEL in sensitizing and eliciting contact allergic reactions to MI. With the detection level of 0.05 ppm this is the case.

Even laundry detergents spiked with artificially high concentrations of MI up to 1000 ppm can be used safely as there are no detectable levels of MI in the washed clothes. This information is important for the consumer as the concentration of MI is not labeled on the package and in addition is still safe in case of overdosing the detergent [7–9].

Currently we notice that the discussion about MI has become very emotional even among scientists and is also incriminating the whole chemical group of isothiazolinones, especially benzisothiazolinone (BIT) and octylisothiazolinone (OIT). But sensitization to MI does not show an immunological cross reaction to BIT and OIT [16]. We hope that our findings help to start a more rational debate as consumer products in many fields are still depending on the use of preservatives to ensure microbiological safety for the consumer.

## Conclusion

It is important to discuss the safety of all ingredients in household products which may cause contact allergy but it is also important to have an evidence based rational debate. As for the risk of exposure levels for the consumer to MI in clothes, it can be stated that the use of MI in laundry detergents is safe for the consumer if these products are used according to the instructions in a normal household setting machine wash as MI is not detected in washed clothes.

Furthermore, it is important to see a potential contact allergen in different ways. A potential contact allergen as a preservative should not be banned totally but has to be seen in context of its usage.

## Abbreviations

BIT: benzisothiazolinone
HPLC: high performance liquid chromatography
MI: methylisothiazolinone
MCI: methylchloroisothiazolinone
NOEL: no observed effects level
OIT: octylisothiazolinone
RSD: relative standard deviation
SCCS: Scientific Committee on Consumer Safety of the European Commission
